# Assessment of knowledge, attitude, and practice toward first aid management of choking hazards among Eastern Province Saudi adults: an observational study

**DOI:** 10.3389/fpubh.2024.1376033

**Published:** 2024-06-13

**Authors:** Ashokkumar Thirunavukkarasu, Abdulrahman Raji Alanazi, Abdullah N. Al-Rasheedi, Danah Khalid Alruwaili, Doaa Mazen Abdel-Salam, Nasser Saleh Alriwely, Abdulrahman Fayez J. Alruwaili, Abdulhadi Abdullah Z. Alanazi, Sultan Farhan O. Alruwaili, Abdulaziz Raja R. Alruwaili

**Affiliations:** ^1^Department of Family and Community Medicine, College of Medicine, Jouf University, Sakaka, Saudi Arabia; ^2^Department of Otolaryngology and Head and Neck Surgery, College of Medicine, Jouf University, Sakaka, Saudi Arabia; ^3^College of Medicine, Jouf University, Sakaka, Saudi Arabia; ^4^Department of Public Health and Community Medicine, Faculty of Medicine, Assiut University, Assiut, Egypt; ^5^Medical Service Department in Ministry of Health, Riyadh, Saudi Arabia

**Keywords:** choking, first aid, children, knowledge, attitude, parents, Saudi Arabia

## Abstract

**Background and aim:**

Childhood choking is a global health concern that mainly affects children under the age of 5 years. The parent’s and caretaker’s responsibility is critical in the children’s lives and can potentially influence the result of at-home injuries such as choking. We aimed to assess the knowledge, attitude, practice, and associated factors of first aid management toward choking hazards among Saudi adults from the Eastern Province.

**Methods:**

The present analytical study was carried out among 390 Saudi adults attending different primary health centers in the Eastern Province of Saudi Arabia. We used a standard and validated data research topic tool to assess knowledge, attitude, and practice. Spearman’s correlation was applied to determine the correlation between each section, while binomial logistic regression analysis was applied to identify the associated factors.

**Results:**

We observed knowledge, attitude, and practice scores in 43.3, 38.9, and 36.4% of the participants, respectively. Furthermore, positive correlations between knowledge and attitude (rho = 0.42, *p* = 0.001), between knowledge and practice (rho = 0.57, *p* = 0.001), and between attitude and practice (rho = 0.41, *p* = 0.001) were revealed in our survey. The knowledge of the participants was significantly higher with the age group of 30–40 years [adjusted odds ratio (AOR) = 3.67 (1.94–4.65), *p* = 0.001] and participants who received training in first aid management [AOR = 1.64 (1.12–2.49), *p* = 0.037]. This study found that males [AOR = 0.36 (0.21–0.63), *p* = 0.001] and those working in the private sector [AOR = 0.61 (0.31–0.87), *p* = 0.018] had significantly lower attitudes.

**Conclusion:**

Our results underscore the importance of continuous health education initiatives and training courses at primary health care centers regarding first aid management of choking hazards to improve awareness and practices. Furthermore, we recommend prospective multicenter studies to address region-specific knowledge gaps.

## 1 Introduction

The term “choking” refers to the interruption of breathing that occurs when food or other foreign objects block the airway (1, 2). It is also known as foreign body airway obstruction (2). It is a medical emergency that requires immediate assistance from anyone nearby to save the victim’s life (3). Childhood choking is a global health concern that particularly affects children under the age of 5, and it is primarily attributed to the tendency of young children to explore their surroundings by placing objects into their mouths (4, 5). Food, coins, toys, and balloons are the most common choking hazards that children experience when eating and playing (6, 7). Choking is a prevalent cause of injury-related morbidity and mortality, particularly among children under the age of 5 years, and it ranks as the fourth most frequent global cause of death in children (8).

Choking stands out as a leading factor behind fatalities in children below the age of four in the United States, constituting the primary reason for mortality among those under 6 years old in a home setting (9). Coughing, speaking and/or breathing difficulties, grasping the throat, and a cyanotic appearance are the classic signs of choking (10). According to an experimental study conducted in 2018 using an internet-based educational video, 202 parents of children aged 6 months to 4 years showed a considerable positive attitude toward the management of choking (2). Recent studies in the Kingdom of Saudi Arabia (KSA) highlight the significance of unintentional injuries among children, including choking hazards (11, 12). For instance, in assessing home accidents and contributing factors among under-5 children in KSA by Alamr F et al., airway obstruction (choking) leading to asphyxia (27.6%) was identified as a significant cause of accidents among their study participants (11). Furthermore, findings from neighborhood countries such as the United Arab Emirates indicate that Choking hazards are significantly high in this region (13). In addition to preventive measures, parents must be aware of first aid procedures when a choking incident occurs. For children over the age of 1 year, choking can be treated with basic life support techniques that combine an abdominal thrust and a back blow (14). The Heimlich maneuver, also known as abdominal thrust, is a more successful intervention technique for clearing airway obstruction caused by foreign bodies (15). Moreover, various organizations, such as the Red Cross Society and the American Heart Association (AHA), have developed guidelines for educating people on preventing and managing choking incidents (14, 16). These organizations have proposed several teaching mechanisms, including first-aid management procedures for choking (17).

A parent’s responsibility is critical in the child’s lives, and it can potentially influence the outcome of at-home injuries and preventive measures (18, 19). The vast majority of choking incidents in children happen while under adult supervision, highlighting a deficiency in people’s knowledge regarding child supervision (20). Thus, a significant number of children do not receive assistance during choking incidents because potential helpers are apprehensive about providing aid without adequate knowledge or training on how to assist children in such situations (8). A substantial amount of research on preventing childhood injuries indicates that educating parents and caregivers can lower the risk of childhood trauma (21).

Between 1982 and 1983, Israel implemented a choking prevention campaign aimed at reducing choking incidents throughout the country (21). The initiative utilized a combination of mass media and clinician offices, resulting in a significant 35% decrease in choking incidents (8). Moreover, a study carried out in 2023 to assess Palestinian mothers regarding knowledge, attitude, and practice about choking hazards shows that younger maternal age, higher socioeconomic class, higher level of education, and occupation status are all significant determinants of a better response among the maternal group (12). Therefore, it is essential for parents and caregivers to be aware of things that cause choking hazards, and they must know about ways to reduce the risk and be competent enough to handle the event of choking (22).

According to the studies cited, educational initiatives aimed at lowering the occurrence of choking-related injuries and fatalities can be successful and effective (23, 24). Despite the importance of evaluating knowledge, attitudes, and practices related to decreasing choking hazards among young children, there is a noticeable lack of comprehensive research in KSA that examines these factors. Consequently, this study intends to bridge this research gap by exploring the level of knowledge among Saudi adults, understanding their attitudes and beliefs regarding choking hazards, and assessing the methods they employ to reduce these risks. Thus, this study has a more significant role in the prevention and importance of timely management of choking hazards among children. Hence, we aimed to assess the knowledge, attitude, practice, and associated factors of first aid management toward choking hazards among Saudi adults from the Eastern Province.

## 2 Participants and methods

### 2.1 Study description

The current cross-sectional investigation took place between June 2023 and December 2023. This survey was conducted among people attending different primary health centers (PHCs) in Dammam city, KSA. Dammam is one of the cities in the eastern region of the KSA. There are 119 PHCs in this region, serving a population of 1,329,000 (25). In the KSA, the PHC provides the first level of care for the community, including health promotion activities for the general population. We included Saudi citizens aged 18 years and over who were willing to participate in the survey, and we excluded those who were mentally unstable, those under 18 of age, individuals who were unwilling to participate, and those who were not present during the data research topic period.

### 2.2 Sample size estimation and sampling method

We used the World Health Organization (WHO) sample size formula (*n* = *z*^2^*pq* /*e*^2^) to estimate the required number of adults to participate in the present survey (26). During estimation, we considered 50% of the expected adequate knowledge (*p*), *q* = 1-*p*, 95% of the confidence interval (*z* = 1.96), and 5% of the margin of error (d). After carefully calculating the above-stated values, we concluded that 384 participants were required for the survey (rounded to 390). These required participants were selected from 10 randomly selected PHCs. We applied a nonprobability consecutive sampling method to obtain the required number of participants. Furthermore, we restricted our data research topic per day to a maximum of 10 participants to ensure that the data were collected over a period of time.

### 2.3 Data research topic procedure

We received ethical approval from the Jouf University bioethics committee (approval no: 06–09-44, dated – May 21, 2023). Next, we obtained permission from the concerned authorities to distribute the questionnaire to the people attending the PHCs. After a briefing about the study and obtaining informed consent, we used the validated Arabic version of the data research topic form. The data research topic tool (survey questionnaire) was prepared by a panel of experts from the Ear, Nose, and Throat (ENT), family medicine, emergency, and nursing departments based on focus group discussions and published works of literature (17, 27, 28). The questionnaire consists of four sections; the first section asks about participants’ sociodemographic characteristics. The next three sections asked about the participants’ knowledge, attitude, and practice toward choking and first aid management for choking. In the knowledge (7 items) and practice (8 items) sections, participants answered in the best answer format. The correct answers were marked with 1 point, and the incorrect answers were marked with 0 points. We sum up total scores, and the mean score for knowledge and practice domains is calculated. For the section related to attitude (comprising seven items), participants responded using a 5-point Likert scale, where choices spanned from strongly agree (5 points) to strongly disagree (1 point). Similar to the knowledge and practice domains, we aggregated scores for attitude domain and calculated mean value. Furthermore, we converted each domain score into 100% and classified them into low (up to 50% of the total score), medium (51 to 75% of the total score), and high (exceeding 75% of the total score). The prepared questionnaire was tested for validity and reliability during the pilot study. The Cronbach’s alpha values for the knowledge, attitude, and practice sections were 0.81, 79, and 0.88, respectively.

### 2.4 Statistical analysis

We used the Statistical Package for Social Sciences (SPSS IBM, V.23) for data entry and analysis. We depicted the descriptive findings of the sociodemographics and participants’ responses in the knowledge, attitude, and practice section as frequencies, proportions, mean and standard deviation (SD). Our data did not meet the normality assumption identified through appropriate tests. Hence, we applied Spearman’s (nonparametric) test to obtain the correlation value (rho) to find the strength and direction of the association. Finally, we combined the low and medium and compared them with the high categories using binomial logistic regression analysis. The significant (*p*-value) value was set at 0.05. All the appropriate applied statistical tests were two-tailed.

## 3 Results

During the data research topic period, we contacted 447 Saudi adults to achieve the estimated sample population (response rate: 87.2%). Among the 390 participants under study, the majority (41.0%) were within the age bracket of 31 to 40 years, were female participants (62.8%), were working in the government sector (67.7%), the majority (80.5%) were currently married, 74.9% were studies university and above, and 72.3% had income above 7,000 SAR. Regarding the number of children, more than half (54.4%) had three or fewer. Furthermore, more than half (58.7%) of the participants never attended any training program related to the first aid management of choking hazards (Table 1).

**Table 1:** Sociodemographic characteristics of the study population (*n* = 390).

Variable	Frequency	Proportion
Age group:		
Less than 30	102	26.2
30 to 40	160	41.0
41 and above	128	32.8
Gender		
Female	245	62.8
Male	145	37.2
Marital status:		
Currently married	314	80.5
Single	76	19.5
Occupation:		
Government	264	67.7
Private	32	8.2
Self employed	22	5.6
Unemployed	72	18.5
Level of education:		
Up to high school	98	25.1
University and above	292	74.9
Monthly income		
Less than 5,000 SAR	69	17.7
5,000 to 7,000 SAR	39	10.0
More than 7,000 SAR	282	72.3
Number of children		
≤ 3	212	54.4
>3	178	45.6
Previous training in first aid management		
No	229	58.7
Yes	161	41.3

Among the participants, 143 (36.7%) responded correctly to the universal sign of choking, 243 (62.3%) to factors leading to choking among preschoolers, 182 (46.7%) to potential choking hazard items, 301 (77.2%) to golden time for providing choking first aid, 328 (84.1%) to symptoms of complete airway obstruction, 322 (82.6%) to symptoms of partial airway obstruction, and 150 (38.5%) responded correctly to choking induced by aspiration of fluids. The mean ± SD of the knowledge domain of the participants was 4.28 ± 1.54 (Table 2).

**Table 2:** Participants correct responses in knowledge category (*n* = 390).

Items	Frequency	Percentage
The universal sign of choking	143	36.7
Elements contributing to choking incidents in preschoolers	243	62.3
Potential choking hazard item	182	46.7
Golden time for administering first aid, choking	301	77.2
Symptoms of complete airway obstruction	328	84.1
Symptoms of partial airway obstruction	322	82.6
Choking induced by aspiration of fluids	150	38.5
Overall score (mean ± standard deviation [SD])	4.28 ± 1.54	

Most participants (85.6%) agreed that choking should require urgent care. Similarly, 84.4% agreed that all must be familiar with first aid management for choking. When asked if choking may not lead to a fatal or life-threatening situation even if not managed, 48.2% strongly disagreed, and 31.5% strongly agreed. A total of 40.5% agreed that choking can be managed at school (without taking it to the hospital). Regarding not providing choking first aid without knowledge, almost 46% agreed on the fact that if first aid for choking is not provided during critical times, it may lead to fatalities. The mean ± SD of the attitude domain of the participants was 19.46 ± 3.81 (Table 3).

**Table 3:** Participants responses in attitude section (*n* = 390).

Parameters	Strongly disagree *n* (%)	Disagree *n* (%)	Neutral *n* (%)	Agree *n* (%)	Strongly agree *n* (%)
Choking should need immediate management	5 (1.3)	46 (11.8)	5 (1.3)	327 (83.8)	7 (1.8)
All must be familiar with choking first aid management	4 (1)	48 (12.3)	5 (1.3)	329 (84.4)	4 (1)
Choking may not lead to a fatal/life-threatening situation even if not managed	188 (48.2)	31 (7.9)	20 (5.1)	28 (7.2)	123 (31.5)
Choking can be managed at school (without taking to the hospital)	53 (13.6)	63 (16.2)	98 (25.1)	158 (40.5)	18 (4.6)
We need to sweep fingers into the throat of the choked child blindly and then take the victim to a hospital.	124 (31.8)	40 (10.3)	87 (22.3)	67 (17.2)	72 (18.5)
You should not administer choking first aid without understanding	39 (10)	109 (27.9)	88 (22.6)	127 (32.6)	27 (6.9)
If first aid for choking is not provided within critical time, it may lead to fatality	16 (4.1)	147 (37.7)	46 (11.8)	179 (45.9)	2 (0.5)
Overall score (mean ± SD)	19.46 ± 3.89				

Regarding practices related to first aid management of choking hazards, less than 50% of the correct answers were observed for all items, except for the responses related to the participants’ practice of child choking while eating but who could speak (55.9%). The mean ± SD of the practice domain of the participants was 3.54 ± 1.43 (Table 4).

**Table 4:** Participants responses in practice section (*n* = 390).

Practice questions	Correct answer	
	Frequency	%
Your action for a choking child experiencing difficulty breathing and speaking, with a completely obstructed airway and no visible food (Step 1).	92	23.6
What will you do next if your initial procedure (previous step) is failed (Step 2)?	143	36.7
No. of times to repeat the procedure (Step 1 and Step 2)	60	15.4
Location of the body to perform the procedure	48	12.3
Your action when facing a child suddenly choking during mealtime, experiencing difficulty breathing and unable to talk, with a complete airway obstruction where a foreign body is visible and accessible.	147	37.7
Your action if the child is choking and coughing	155	39.7
Your action if the child is choking while eating but could speak	218	55.9
Your action if the choking in your presence but cannot cough, talk, or breathe	128	32.8
Overall score (mean ± SD)	3.54 ± 1.43	

**Figure 1:**
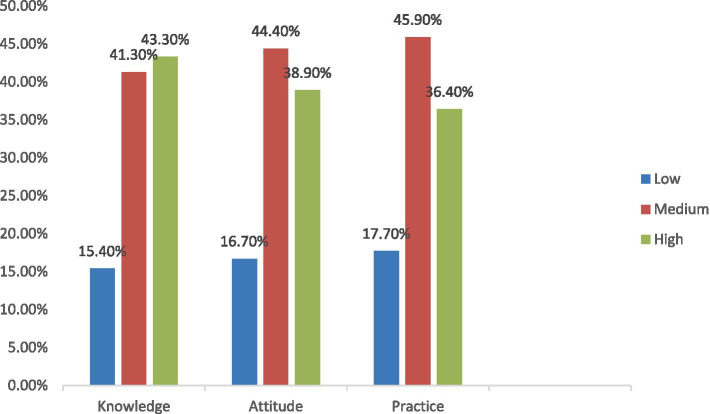
KAP categories.

We observed high knowledge, attitude, and practice scores in 43.3, 38.9, and 36.4% of the 390 studied participants, respectively (Figure 1: KAP Categories).

The present study observed a positive correlations between knowledge and attitude (rho = 0.42, *p* = 0.001), between knowledge and practice (rho = 0.57, *p* = 0.001), and between attitude and practice (rho = 0.41, *p* = 0.001) (Table 5).

**Table 5:** Spearman’s correlation analysis between KAP scores.

Variable	rho* / *p*-value
Knowledge – Attitude	0.42 (0.001)
Knowledge – Practice	0.57 (0.001)
Attitude – Practice	0.41 (0.001)

* Spearman’s rank correlation coefficient, ** significant at 0.05 level (2-tailed).

The knowledge of the participants was significantly higher with the age group of 30–40 years [ref: less than 30 years, AOR = 3.67 (1.94–4.65), *p* = 0.001] and participants who received training in first aid management [ref: no training received, AOR = 1.64 (1.12–2.49), *p* = 0.037]. The present study found that participants with similar characteristics had significantly higher scores in attitude and practice aspects. However, this study found that males [ref: females, AOR = 0.36 (0.21–0.63), *p* = 0.001] and those working in the private sector [ref: government, AOR = 0.61 (0.31–0.87), *p* = 0.018] had significantly lower attitudes (Table 6).

**Table 6:** Multivariate analysis on KAP subscales (low/medium vs. high) and its association with participants background traits (*n* = 390).

Variables	Total	Knowledge				Attitude				Practice			
		Low/Mediu *n* = 221	High *n* = 169	Adjusted OR (95% CI) *	*p*-value**	Low/Medium *n* = 238	High *n* = 152	Adjusted OR (95% CI) *	*p*-value**	Low/ Medium *n* = 248	High *n* = 142	Adjusted OR (95% CI) *	*p*-value**
Age (in years)													
Less than 30	102	45	57	Ref		52	50	Ref		74	28	Ref	
30 to 40	160	130	30	3.67 (1.94–4.65)	0.001	121	39	2.72 (1.91–4.15)	0.001	88	72	1.69 (1.10–2.33)	0.006
41 and above	128	46	82	0.76 (0.44–1.33)	0.337	65	63	0.91 (0.52–1.59)	0.736	86	42	0.76 (0.42–1.38)	0.370
Gender													
Female	245	126	119	Ref		162	83	Ref		153	92	Ref	
Male	145	95	50	1.64 (0.94–2.85)	0.082	76	69	0.36 (0.21–0.63)	0.001	95	50	1.09 (0.65–1.85)	0.722
Marital status:													
Married	314	178	136	Ref		191	123	Ref		193	121	Ref	
Single	76	43	33	1.93 (1.04–3.59)	0.037	47	29	1.77 (0.95–3.31)	0.075	55	21	1.27 (0.68–2.39)	0.457
Occupation:													
Government	264	150	114	Ref		166	98	Ref		164	100	Ref	
Private	32	16	16	0.62 (0.26–1.45)	0.265	15	17	0.61 (0.31–0.87)	0.018	17	15	0.60 (0.27–1.33)	0.201
Self employed	22	9	13	0.39 (0.14–1.01)	0.074	18	4	2.71 (0.82–6.48)	0.101	19	3	3.22 (0.87–6.11)	0.079
Unemployed	72	46	26	0.84 (0.62–2.11)	0.865	39	33	0.86 (0.36–2.03)	0.723	48	24	0.57 (0.24–1.33)	0.191
Education status:													
Up to high school	98	52	46	Ref		59	39	Ref		68	30	Ref	Ref
University and above	292	169	123	0.87 (0.49–1.54)	0.634	179	113	1.32 (0.76–2.32)	0.328	180	112	0.81 (0.47–1.41)	0.466
Income (SAR)													
Less than 5,000	69	41	28	Ref		36	33	Ref		51	18	Ref	Ref
5,000 to 7,000	39	17	22	0.54 (0.39–1.21)	0.238	27	12	2.26 (0.80–4.37)	0.123	25	14	0.55 (0.20–1.49)	0.241
More than 7,000	282	163	119	0.73 (0.57–1.36)	0.283	175	107	1.15 (0.48–2.73)	0.759	172	110	0.47 (0.19–1.13)	0.090
Number of children													
≤ 3	212	121	91	Ref		132	80	Ref		134	78	Ref	Ref
>3	178	100	78	1.17 (0.73–1.88)	0.506	106	72	1.02 (0.64–1.67)	0.939	114	64	1.21 (0.77–1.89)	0.403
Previous training in first aid management													
No	229	122	107	Ref		106	55	Ref		103	58	Ref	
Yes	161	99	62	1.64 (1.12–2.49)	0.037	132	97	1.93 (1.22–3.08)	0.005	145	84	1.85 (1.18–2.90)	0.007

* Adjusted with other variables of the study. ** *p*<0.05 is a significant factor.

## 4 Discussion

Prevention and reduction of illness and death related to choking hazards among children can be achieved with the adequate knowledge and skills of their parents (2, 6). Basic life support training for parents about these life-threatening conditions will enhance the chances of children’s survival under 5 years of age (29). Hence, the current research aimed to assess the knowledge, attitude, and practice toward first aid management of choking hazards. Concerning the knowledge of the participants in the present study, 43.3% of the studied participants had good knowledge about the first aid management of choking hazards. The present study revealed that 62.3, 77.2, 84.1, and 82.6% of the participants responded correctly to factors that led to choking among preschoolers: golden time for providing choking first aid, complete airway obstruction symptoms, and partial airway obstruction symptoms, respectively. In contrast, fewer than 50% of the correct answers were observed for all the practice questions, except for the responses related to the participants’ practice with the child choking while eating but being able to speak (55.9%). Therefore, the present study revealed a gap between the knowledge and practice of parents regarding the first aid management of choking hazards. This finding agreed with research conducted by Asmar et al. in 2023, which revealed that 72.9% of mothers exhibited good knowledge, while 75.9% of the participants demonstrated poor practices in relation to first aid measures for choking (30).

The knowledge of first aid management of choking hazards in the present study was high, medium, and low among 43.3, 41.3, and 15.4% of the participants, respectively. This finding is slightly higher than that reported in other surveys (31, 32). A survey conducted by Eldosoky et al. exhibited that mothers answered an average of 11 out of 29 knowledge questions correctly (33). Furthermore, a study by Suguna in India found that 48.7% of mothers had average knowledge of first aid in domestic accidents (34). On the other hand, research conducted by Harere et al. in Saudi Arabia assessing parents’ and caregivers’ knowledge of first aid for common emergency conditions in children revealed that 94.4% of the studied sample had inadequate knowledge (35). The variation in knowledge of choking hazards and first aid management across different studies could be due to differences in study populations, settings, and tools used to assess knowledge.

The attitudes of the participants toward first aid management for choking hazards in the present study were high and medium among 38.9 and 44.4% of the participants, respectively. A mere 16.7% of the participants in this study exhibited a low attitude toward first aid management. This result is higher than that shown in other studies (36). This difference between the present study and other studies regarding knowledge and attitude toward first aid measures could be attributed to the differences in the studied samples’ sociodemographic and socioeconomic characteristics.

Concerning the practice of first aid management of choking hazards in this study, less than 50% of the correct answers were observed for all the practice questions, except for the responses related to the participants’ practice with the child choking while eating but being able to speak (55.9%). This might be linked to their perception of the low vulnerability of children to injuries, and they believe that they can ensure their child’s safety through close monitoring. Another explanation of this finding is that, even though the parents had good knowledge and attitudes in this study, this did not lead to the same proportion of practice. This may have been because of the absence of real-time situations or regular training that might have needed them to practice their choking first aid skills. A study carried out by Asif et al. revealed that 25% of mothers encountered a choking child, 9.5% of whom positioned themselves behind the child, encircling the child’s chest (36). Another study conducted at Al-Khobar city in KSA revealed that 80.8% did not have knowledge about cardiopulmonary resuscitation, which is very important in handling choking (37). Concerning first aid practices for choking, Zedain et al. showed that more than half of the mothers performed correct actions, such as giving mouth breathing to the child anterior fontanel, hanging a choking child upside down by the feet, or smelling the affected child perfume (32). With respect to mothers’ the practice of first aid in Qassim, KSA, 43.2% of mothers showed appropriate practice in dealing with choking (38). However, a different picture was shown in the study perfromed by Midani et al. in the UAE, who indicated that 80.6% of the participants knew how to deal with choking (39). These differences in the practice of first aid measures may be attributed to variations in educational background and attendance at first aid training courses. Parents’ knowledge, attitude, and practice were significantly associated with their age in this study. This indicates that knowledge level, attitude, and practice increase as age increases, which may be associated with experiences with older children (40, 41). The present study revealed that parents aged from 30 to 40 years were more knowledgeable and had better attitudes and practices compared to those aged less than 30 years.

Similar to other studies, we revealed that parents’ previous training was substantially related to their knowledge, attitude, and practice of first-aid management of choking hazards (42–44). Individuals with prior experience in first-aid training exhibited approximately twice the level of knowledge compared to those without such training in this study. Furthermore, these parents had a better attitude and practiced two times more than those who did not receive previous training. The present study revealed that males had less attitude toward first aid management of choking hazards than females, in agreement with a study conducted by Adere et al. (45). This can be explained by the fact that females, particularly mothers, consistently maintain direct contact with their children at home, especially during the infancy and preschool stages. Mothers have a great deal of responsibility to have proper knowledge and practice about domestic accidents and first aid measures, as well as take preventive measures to ensure the safety of the home environment in addition to close supervision of their children (46). The current study revealed that parents working in the private sector had less attitude toward first aid management for choking hazards compared to those working in the government sector, which is in agreement with the findings of a study conducted in Egypt (33). This can be explained by the fact that those working in the government sector receive higher wages. This allows for more access to resources and medical care among parents in the government sector, which is associated with an increase in knowledge and attitude toward first aid measures.

There are some limitations encountered in the present study: the first, temporality cannot be established because of the cross-sectional design. A prospective survey can be conducted in future research to find stronger associations. Second, because of the self-administered nature of the questionnaire, bias related to participants’ level of understanding can arise. Furthermore, the scarcity of comparable studies restricted the ability to make comprehensive comparisons with existing research. Finally, we assessed the knowledge gap about choking hazards in the Eastern Province of the KSA. Hence, the findings of the current research cannot be generalized to the entire KSA due to prevailing sociocultural variations across different regions.

## 5 Conclusion

We found that more than half of the participants had either low or medium knowledge, attitude, and practice regarding first aid management of choking hazards. Age and previous training in first aid management significantly influenced the participants’ knowledge, attitude, and practice scores. Furthermore, males and those working in the private sector had less attitude toward the first aid management of choking hazards. This finding highlights the importance of continuous health education programs and training courses at primary health care centers regarding first aid management of choking hazards for Saudi adults to improve their awareness and practices. In addition, incorporating first aid measures into school curricula may be beneficial.

## Data availability statement

The raw data supporting the conclusions of this article will be made available by the authors, without undue reservation.

## Ethics statement

The studies involving humans were approved by Local Committee of Bioethics (LCBE), Jouf University. The studies were conducted in accordance with the local legislation and institutional requirements. The participants provided their written informed consent to participate in this study.

## Author contributions

AT: Conceptualization, Data curation, Formal analysis, Software, Validation, Writing – original draft. ARA: Conceptualization, Data curation, Formal analysis, Validation, Writing – original draft. AA-R: Conceptualization, Funding acquisition, Methodology, Validation, Writing – original draft. DA: Conceptualization, Data curation, Methodology, Writing – original draft. DA-S: Conceptualization, Formal analysis, Software, Visualization, Writing – original draft. NA: Conceptualization, Investigation, Validation, Writing – review & editing. AFJA: Conceptualization, Data curation, Methodology, Writing – review & editing. AAZA: Conceptualization, Data curation, Methodology, Writing – review & editing. SFOA: Data curation, Software, Validation, Writing – review & editing. ARRA: Data curation, Validation, Writing – review & editing.
